# Clarifying the Adsorption of Triphenylamine on Au(111): Filling the HOMO–LUMO Gap

**DOI:** 10.1021/acs.jpcc.1c08877

**Published:** 2022-01-17

**Authors:** Teng Zhang, Pamela H. W. Svensson, Iulia Emilia Brumboiu, Valeria Lanzilotto, Cesare Grazioli, Ambra Guarnaccio, Fredrik O. L. Johansson, Klára Beranová, Marcello Coreno, Monica de Simone, Luca Floreano, Albano Cossaro, Barbara Brena, Carla Puglia

**Affiliations:** † School of Integrated Circuits and Electronics, MIIT Key Laboratory for Low-Dimensional Quantum Structure and Devices, Beijing Institute of Technology, 100081 Beijing, China; ‡ Department of Physics and Astronomy, Uppsala University, P.O. Box 516, SE-751 20 Uppsala, Sweden; § Department of Chemistry, Pohang University of Science and Technology (P.O.STECH), 37673 Pohang, Republic of Korea; ∥ Department of Chemistry, Sapienza Università di Roma, P.le A. Moro 5, 00185 Roma, Italy; ⊥ IOM-CNR, Laboratorio TASC, Sincrotrone Trieste, Basovizza, 34149 Trieste, Italy; # ISM-CNR, Istituto di Struttura della Materia, 85050 Tito Scalo (Pz), Italy; ¶ Division of Applied Physical Chemistry, Department of Chemistry, KTH Royal Institute of Technology, 10044 Stockholm, Sweden; ∇ Sorbonne Université, UMR CNRS 7588, Institut des Nanosciences de Paris, F-75005 Paris, France; ○ Elettra-Sincrotrone Trieste S. C. p. A., Strada Statale 14, km 163.5, Basovizza, 34149 Trieste, Italy; ⧫ FZU—Institute of Physics of the Czech Academy of Sciences, 18221 Prague, Czech Republic; †† Department of Chemical and Pharmaceutical Sciences, University of Trieste, 34127 Trieste, Italy

## Abstract

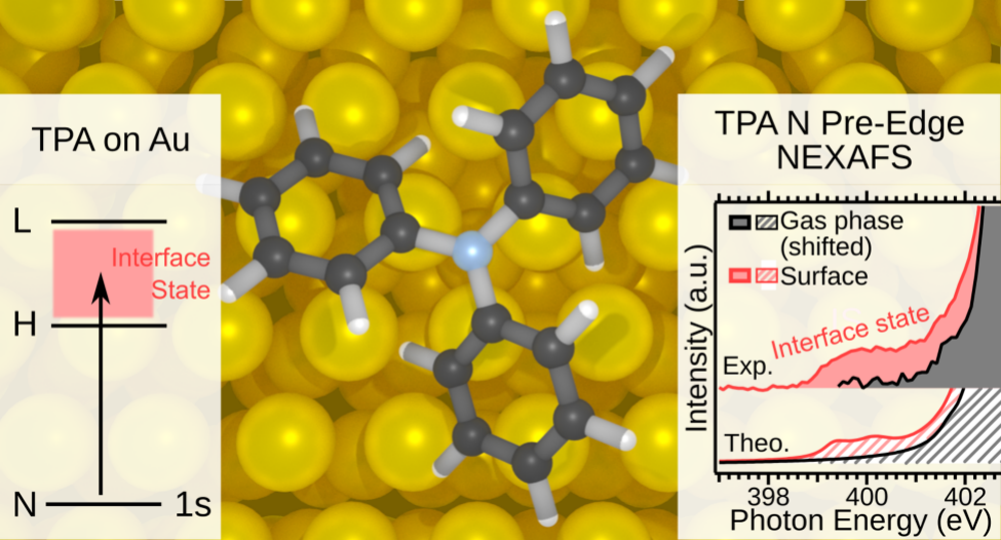

In this article, we analyze the electronic structure modifications of triphenylamine (TPA), a well-known electron donor molecule widely used in photovoltaics and optoelectronics, upon deposition on Au(111) at a monolayer coverage. A detailed study was carried out by synchrotron radiation-based photoelectron spectroscopy, near-edge X-ray absorption fine structure (NEXAFS) spectroscopy, scanning tunneling microscopy (STM), and ab initio calculations. We detect a new feature in the pre-edge energy region of the N K-edge NEXAFS spectrum that extends over 3 eV, which we assign to transitions involving new electronic states. According to our calculations, upon adsorption, a number of new unoccupied electronic states fill the energy region between the highest occupied molecular orbital (HOMO) and the lowest unoccupied molecular orbital (LUMO) of the free TPA molecule and give rise to the new feature in the pre-edge region of the NEXAFS spectrum. This finding highlights the occurrence of a considerable modification of the electronic structure of TPA. The appearance of new states in the HOMO–LUMO gap of TPA when adsorbed on Au(111) has crucial implications for the design of molecular nanoelectronic devices based on similar donor systems.

## Introduction

In recent years, organic semiconductors and metal interfaces have been the focus of a large number of studies aiming to conveniently engineer innovative materials with tunable energy levels that can be exploited in molecular- and nanoelectronics. In supramolecular chemistry, novel materials with specific properties are obtained by combining different molecular units into molecular assemblies adsorbed on surfaces. To design these materials, it is clearly fundamental to know the characteristics of the single complexes and to investigate the modifications these complexes undergo, in structure and properties, when they are adsorbed on a substrate. Of high interest in this field are the π-conjugated molecules adsorbed onto non-reactive metal surfaces, such as noble metals, which are often used in optoelectric and photovoltaic devices. In this respect, it would be natural to expect that the adsorption of π-conjugated molecules on these surfaces would be characterized by a weak interaction or, in case, by an interaction of strength between physisorption and weak chemisorption.^1^ In the present work, we show that a combination of spectroscopic methods and theoretical calculations represents a unique and powerful tool to investigate adsorbate systems. We focus on the adsorption of triphenylamine (TPA) on the Au(111) surface: besides being a model case for the investigation of the molecular interaction with low-reactive metallic surfaces, this system is also interesting because Au is often used as a contact material in many types of devices. In the present study, we show evidence of a significant molecule/substrate interaction, practically abating the molecular energy gap and questioning the suitability of employing the studied materials in electronic devices.

TPA is an organic molecule whose shape can ideally be derived from ammonia by substituting the three hydrogens by phenyl groups. The three N–C bonds of TPA constitute a planar “core” structure, where the phenyl rings arrange in space providing its typical non-planar and propeller-like shape. TPA is intensively studied because of its excellent electron-donating and hole-transport properties. Owing to these characteristics, TPA and its derivatives have successfully been implemented in optoelectronic devices like OLEDs, in photovoltaics as donors, in organic dye-sensitized solar cells (DSSCs), and as hole transport materials in perovskite solar cells.^2−5^ Our recent publication has provided comprehensive information on the electronic structure of the isolated TPA, with a detailed characterization of the molecular HOMO (highest occupied molecular orbital) and LUMO (lowest unoccupied molecular orbital), explaining that the electron-donating characteristics of TPA can be ascribed to the N lone pair contribution to the molecular HOMO.^6^ As already mentioned, the interaction between the molecule and a surface can play an important role in the operation of molecular electronic devices. In DSSCs, for example, the charge generation and separation, which are crucial steps for light-harvesting, happen at the interfaces between molecules and substrates or between molecules of donor and acceptor characters. Our spectroscopic results on the adsorption of TPA on Au(111) indicate a significant change in the electronic structure of TPA upon adsorption, highlighting a significant interaction between the molecule and Au(111).

The characterization of the adsorption system has been performed by means of photoelectron spectroscopy (PES) and near-edge X-ray absorption fine structure (NEXAFS). Interestingly, the N K-edge NEXAFS spectrum of TPA/Au(111) reveals an extra feature in the pre-absorption edge, which is more intense and extends over a broader energy region compared to a similar pre-edge peak found in the gas-phase spectrum,^6^ suggesting that new electronic states are formed between the HOMO and the LUMO of the adsorbed molecule. Our theoretical characterization shows that the new states contain a small contribution from the N atom, and a larger contribution from the C and Au atoms and fill up the molecular HOMO–LUMO gap, which is one of the most important parameters directly connected to the operation of electronic and photovoltaic devices. As we can infer from our ab initio density functional theory (DFT) calculations, these states arise in the adsorbate system due to the interaction between the molecule and the surface, thus suggesting that the feature in the N K-edge NEXAFS is mainly an initial state effect, that is an effect not due (or at least not only due) to the electron excitation process.

## Methods

### Experimental Methods

The experiments were carried out at the ALOISA and at the Material Science beamlines of Elettra. Au(111) single crystals were cleaned by repeated cycles of Ar^+^ sputtering and annealing at 500–630 °C. The Au(111) substrate was kept at room temperature during the molecular depositions and during the measurements. The TPA powder, purchased from Sigma-Aldrich with 98% purity, was purified further by thermal treatment and then dosed via a leak valve to form the TPA/Au(111) monolayer. The thickness of the TPA films was well reproducible and estimated by the attenuation of the Au 4f PES core lines. During the measurements, the pressure was in the low 10^–10^ mbar range.

The PES measurements were performed at the Materials Science beamline of Elettra, using a Specs Phoibos 150 hemispherical electron energy analyzer. The C 1s and N 1s core level spectra were measured, respectively, with photon energies of 392 and 495 eV at normal emission. The overall resolutions were about 330 meV and 430 meV for C 1s and N 1s, respectively, estimated from the width of the Fermi edge of the clean Au(111) crystal. Similarly, the overall resolution of the valence spectra, measured with 40 and 100 eV photon energies, was about 150 meV. The binding energy (BE) scales of the valence and core level PE spectra were calibrated with respect to the Fermi level and the Au 4f of the Au(111), respectively.

The C K-edge and N K-edge NEXAFS spectra were measured at the ALOISA beamline of Elettra in the partial electron yield mode by means of a channeltron equipped with a polarizable grid to reject low energy secondary electrons (biases of −230 and −370 V for C and N K-edge, respectively). The energy resolution was set to 80 and 100 meV for the C and N K-edge, respectively. The orientation angle of the photon linear polarization E vector with respect to the surface of the Au(111) substrate was changed from out-of-plane “p-polarization” (p-pol) to in-plane “s-polarization” (s-pol) by rotating the sample around the photon beam direction while keeping a constant grazing angle of 6°. Due to the particular design of the ALOISA manipulator, the p-pol geometry corresponds to beam polarization almost perpendicular to the sample surface (84°). Details about absolute energy calibration and intensity normalization of the NEXAFS spectra can be found in refs (7) and (8). Beam-induced damage was avoided by constantly changing the sample position and checked by N K-edge NEXAFS and C1s PES.^9^

Scanning tunneling microscopy (STM) measurements were performed via an ultrahigh vacuum low-temperature STM system at liquid-helium temperatures (sample temperature at about 5 K). The images were taken in a constant-current scanning mode. The STM tips were obtained by chemical etching from a tungsten (W) wire. Lateral dimensions observed in the STM images were calibrated using an Au(111) lattice.

### Computational Methods

**Figure 1:**
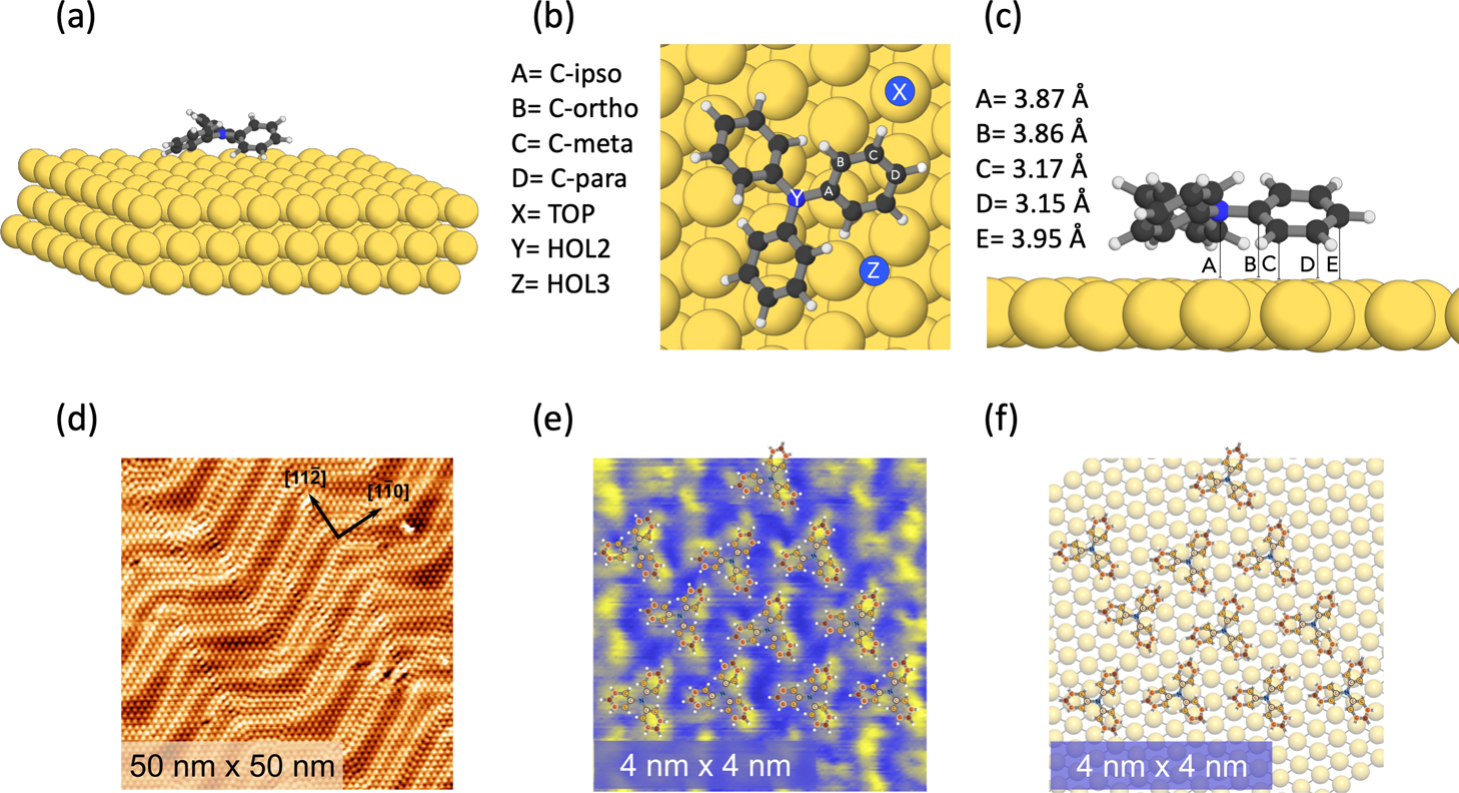
(a) Optimized structure of TPA adsorbed on the Au(111) surface. Au(111) is represented by a three-layer slab. TPA is adsorbed on a HOL2 site, as described in the text. (b) Top view of TPA adsorbed on Au(111) in the HOL2 site. The three sites that were considered for the geometry optimization are indicated in the figure. The TOP site (*X* in the figure), on top of an Au atom of the first layer, the HOL2 site (*Y* in the figure), above an Au atom of the second layer, and the HOL3 site (*Z* in the figure), above an Au atom of the third layer. (c) Side view of TPA adsorbed on Au(111) in the HOL2 site. The distance between some of the C atoms and Au atoms of the substrate is indicated. (d) STM results showing the adsorption of TPA on Au: large area scan (50 nm × 50 nm) where TPA follows the Au(111) herring bone surface reconstruction. Scan parameters are bias = −2 V and *I*_t_ = 100 pA. (e) Small area scan with a high resolution (4 nm × 4 nm). The STM scan parameters are bias = −0.6 V and *I*_t_ = 100 pA. (f) Model illustrating the agreement between the TPA in the HOL2 site and the STM images.

For the structure of TPA and the notation of the different carbons (C-*ipso*, C-*ortho*, C-*meta*, and C-*para*), we adopt the same notation used in ref (6). All the calculations of the adsorbed TPA on Au(111) were performed using the Quantum-ESPRESSO (QE) package^10^ with the PBE functional,^11^ including van der Waals interactions as described by the semiempirical Grimme’s DFT-D3 method.^12^ We have used ultrasoft pseudo-potentials^13^ for N, H, and Au, and norm-conserving pseudopotentials (PPs) for C both in the calculations of the structures and of the spectra.^14^ The plane wave cutoff was 90 eV and the Γ-point *k*-mesh was used. The adsorption system used in the supercell was formed by a TPA molecule on a three-layer Au(111) slab of 9 × 8 atoms (Figure 1a), contained in a monoclinic supercell with a basis of 23.53 Å times 26.48 Å with an angle of 120° in between and with a height of 25 Å. This leaves about 15 Å of vacuum between the adsorbed molecule and the next Au layer. During geometry optimization, the lowest Au layer was kept fixed. After performing a geometry optimization of the TPA molecule in the gas phase, we positioned it on three different high symmetry adsorption sites over the gold layer, named after the position of the central N atom, as indicated in Figure 1b, and performed geometry optimizations of the adsorption system. The total and partial density of states (DOS) and the NEXAFS C K-edge and N K-edge spectra were computed with the *dos* and the *XSpectra* codes within the QE package.^15^ In the NEXAFS calculations, we employed the half core-hole (HCH) method^15^ where an occupation of 0.5 (i.e., 0.5 electrons per electronic state) is chosen to represent the excited C 1s or N 1s electrons. For these calculations, we used ultrasoft PPs for the neutral atoms and constructed GIPAW PPs generated with the *atom* code within the QE package to represent the HCH-excited atoms. To obtain the C partial DOS and the C 1s NEXAFS spectra for TPA in the gas phase, we computed the spectrum of each of the four chemically non-equivalent C atoms in the molecule: C-*ipso*, C-*ortho*, C-*meta*, and C-*para*, as shown in Figure 1a of ref (6); in the case of the adsorbed TPA, we computed the spectra for all the six C atoms on a phenyl ring because, in this case, the two C-*ortho* and C-*meta* become chemically inequivalent due to the rotation of the phenyl rings with respect to the surface (see Figure 1c, side view of the HOL2 configuration).^11^ In order to align, in energy, these spectra with each other, we have computed the C 1s photoelectron energy for each of the six C atoms with the ΔKohn–Sham method and we have manually shifted each spectrum by the relative core level shift.^16^ To facilitate the visualization and the comparison with the experiments, a broadening of 0.2 eV full width at half-maximum was applied to the theoretical spectra. For the simulation of the C 1s and N 1s NEXAFS spectra in gas phase, the *x*, *y*, and *z* components obtained from the calculations were summed up to obtain the total spectrum. For the adsorbed system, we calculated two different spectral components: the in-plane N or C K-edge spectra, that is, the spectra for transitions to orbitals parallel to the surface, as the sum of the *x* and *y* contributions, and the out-of-plane N or C K-edge spectra for transitions to orbitals orthogonal to the surface, directly given by the z contributions.

## Results and Discussion

### Structure of the Adsorbed System

The adsorption sites we tested were a hollow site where the N atom lies on top of an Au atom of the second layer (HOL2, *Y* in Figure 1b), a hollow site where the N atom lies on top of an Au atom of the third layer (HOL3, *Z* in Figure 1b), and an on-top position (TOP, *X* in Figure 1b), where the N atom sits directly on top of an Au atom of the first Au layer. The HOL2 site shown in Figure 1a,b, corresponding to the lowest energy, is used in the rest of the calculations. The HOL2 site shown in Figure 1a corresponds to the adsorption configuration with the lowest total energy, with a difference in adsorption energy of 0.06 eV with respect to the HOL3 site and 0.11 eV with respect to the TOP site. Figure 1b,c shows the top and side views of the relaxed TPA molecule on the HOL2 site. As can be seen from the top view in Figure 1b, the relaxed adsorption geometry is not fully symmetric because the N–C bonds are not precisely oriented along the main symmetry axes of the substrate. The distance of the TPA nitrogen to the average position of the top Au layer is 3.87 Å, while its distance to the Au atom beneath, in the second Au layer, is 6.08 Å. The distances between the lower C-*ortho* in the three phenyl rings from the closest Au atoms on the top layer of the surface vary between 3.16 and 3.19 Å, while the distances of the C-*meta* from the closest Au atoms vary between 3.12 and 3.17 Å. Some of these distances are shown for one of the phenyl rings in Figure 1c. According to our results, the structure of the molecule is only weakly affected by the adsorption, the main difference being that the phenyl rings are rotated by approximately 6° less with respect to the central plane of TPA as compared to the gas phase, making the molecule slightly flatter when adsorbed on the surface.

The theoretical model is compatible with the STM results of TPA/Au(111) measured at 5 K and shown in Figure 1d–f. Moreover, Figure 1d shows that TPA does not alter the Au(111) surface reconstruction and in general follows the herring bone structure. The small area scan in Figure 1e with the model in Figure 1f clearly shows that the STM results are consistent with the HOL2 adsorption site found by the theory.

### C K-Edge NEXAFS

**Figure 2:**
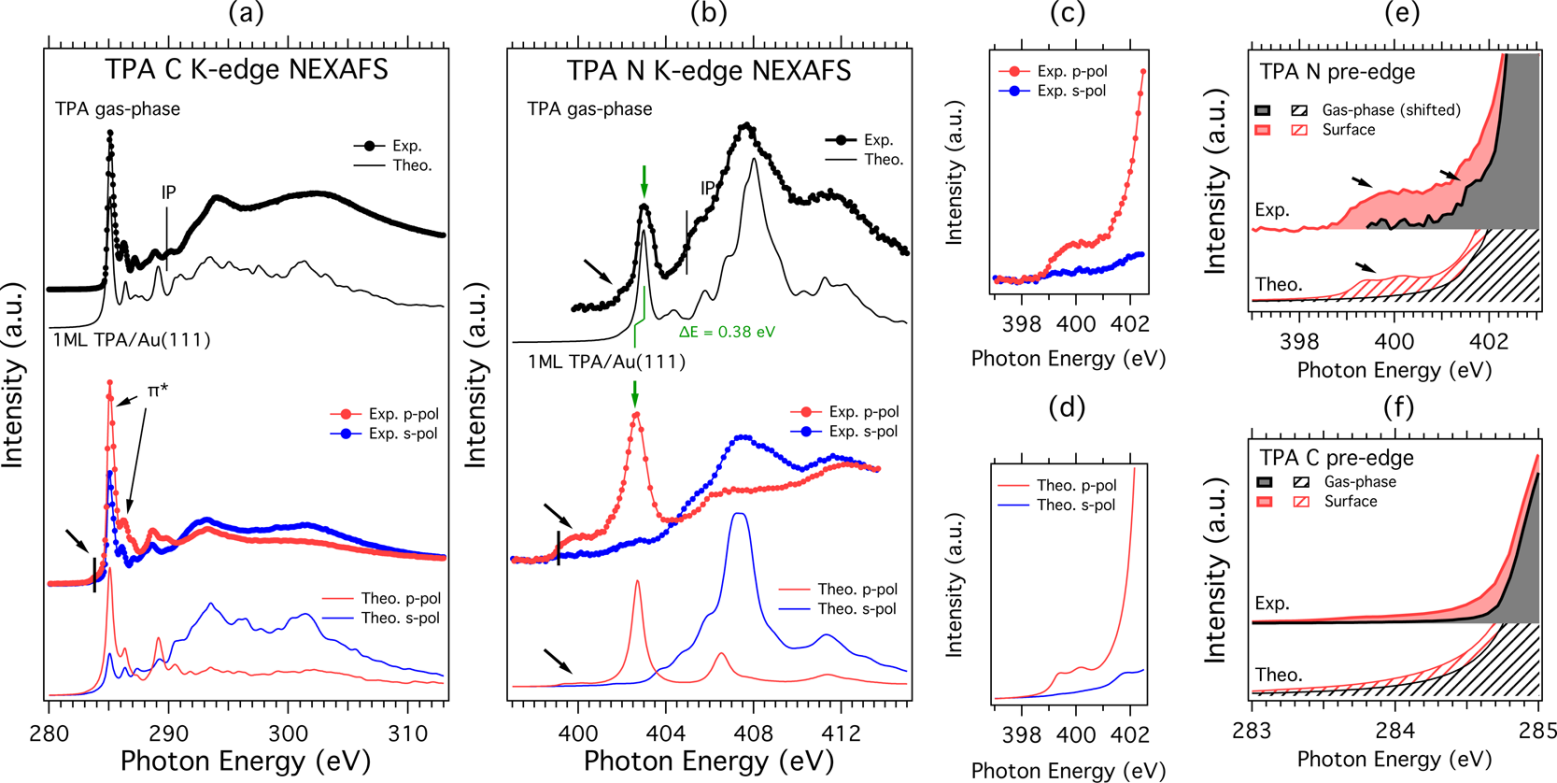
Experimental (thick lines) vs computed (thin lines) spectra of (a) C K-edge NEXAFS and (b) N K-edge NEXAFS of TPA in the gas phase and TPA adsorbed on Au(111). In black are given the gas-phase spectra; in red are the p-pol experimental and theoretical spectra; and in blue are the s-pol experimental and theoretical spectra. The core line ionization potentials are marked with thin black bars (gas phase) and the N 1s and C 1s core line photoemission BE as thick black bars (adsorbate). (c) Zoom in of the N K-edge pre-edge region showing the comparison between experimental p-pol vs s-pol; (d) zoom in of the N K-edge pre-edge region showing the comparison between theoretical p-pol vs s-pol; (e) comparison of the pre-edge peaks in N K-edge NEXAFS between TPA in the gas phase and TPA/Au(111); and (f) comparison of the pre-edge peaks in C K-edge NEXAFS between TPA in the gas phase and TPA/Au(111).

The experimental and theoretical C K-edge NEXAFS spectra of one monolayer TPA/Au(111) are shown in Figure 2a (lower panel) alongside the gas-phase results^6^ (upper panel). The spectra of the adsorbed TPA are generally similar to the gas-phase spectrum. No transition energy shifts between the gas phase and adsorbate are detected and the first resonance peak is at the same photon energy as in the gas phase.^6^

The C spectra shown in Figure 2a are characterized by two main π* peaks at about 285 and 286 eV, well reproduced by the calculations and which have the same origin in the adsorbed as in gas-phase TPA. The first peak at about 285 eV originates from C-*ortho*, C-*meta*, and C-*para* contributions of similar intensities, while the smaller peak at 286.5 eV is due to C-*ipso* contributions.^6^ The spectra for each type of C atoms are reported in the Supporting Information. Due to the molecular structure and adsorption orientation, at energies above 291 eV, we observe some polarization effects that are somewhat stronger in the measured spectra, indicating that in the sample the molecules are slightly more rotated or disordered than in the theoretical model.

Compared to the gas-phase measurement, an additional clearly discernible low-intensity pre-edge feature appears at 284 eV in the spectrum of TPA adsorbed on Au(111), indicated by the arrow in Figure 2a and enlarged in Figure 2f.

For solid-state measurements, polarization-dependent C K-edge spectra (p-pol and s-pol, described in the Experimental Methods section) show a rather pronounced angular dependence, where the π* resonances have stronger intensity in the p-pol/out-of-plane geometry. This intensity variation can be related to the so-called “NEXAFS linear dichroism” and allows one to estimate the molecular orientation with respect to the Au(111) surface. Provided that the interaction with the substrate does not alter the molecular orbital symmetry, the average tilt angle γ for the threefold symmetry substrate can be obtained from the intensity ratio of a π-symmetry resonance in s- and p-polarization as *I*_s_/*I*_p_ ∼ 1/2 tan^2^ γ.^17,18^ The observed degree of NEXAFS dichroism at the C K-edge indicates an average tilt off the surface of about 60° ± 20°, reflecting the orientation of the “propeller-like” phenyl rings.

### N K-Edge NEXAFS

The N K-edge NEXAFS are shown in Figure 2b. As reported in ref (6), the first high intensity π* peak in the spectrum of the gas-phase TPA corresponds to N 1s transitions into the LUMO + 5 molecular orbital, which has significant contribution from the N 2p_*z*_ states. This main peak, which is located at 403.02 eV in the gas phase, is shifted by −0.38 to 402.64 eV in the TPA/Au(111) and is preceded by a pre-edge feature much more intense than that in the gas phase. These results clearly indicate that the electronic structure of TPA is perturbed by the adsorption on the Au(111) surface.

The N K-edge NEXAFS of the adsorbed TPA shows an angular dependence stronger than the C K-edge one. In the p-pol geometry, the π* resonances at energies below 404 eV are greatly enhanced while they are almost undetectable at the s-pol geometry, suggesting that the central plane defined by the N–C-*ipso* bonds of TPA, is flat and aligned parallel to the Au(111) surface. The behavior of the experimental p-pol and s-pol spectra is well reproduced by the simulations both along the in-plane and out-of-plane directions, confirming that the adsorption of the molecules occurs with the central plane parallel to the surface. Importantly, the pre-peak is reproduced both in the out-of-plane (major contribution) and in-plane (minor contribution) spectra, albeit in both cases with a lower intensity than in the experimental spectrum. The fact that the intensity is enhanced in the experimental spectra can be attributed to both dynamic and vibrational effects of the NEXAFS core hole final state, as explained for other adsorbate systems.^19^

In Figure 2e, we highlight a small pre-edge intensity peak in the N K-edge spectrum of gas-phase TPA, which is ascribed to transitions from N 1s to the in-plane orbitals LUMO + 1 up to LUMO + 4 which have low N characters.^6^ On the other hand, when TPA is adsorbed on Au(111), the pre-edge peak extending between 399 and 402 eV is broader than in the gas phase and appears to span over the molecular HOMO–LUMO gap. As shown in enlargements in Figure 2c,d, this N pre-edge feature is sensitive to the incident angle of the light: it is more pronounced at p-pol and less at the s-pol geometry, indicating a π* (out-of-plane) orbital character. The different symmetries of this feature compared to that of the gas phase is a strong indication that this peak is due to the electronic structure modifications induced by the adsorption of TPA on the Au surface. This is also confirmed by the fact that its intensity almost disappears in the NEXAFS spectra acquired for higher molecular coverage (Figure S1), where the interaction with the surface and the monolayer features are attenuated by the outer layers.

Considering that the intensity in a NEXAFS spectrum is related via specific transition rules to a distribution of the unoccupied states, the observation of new features could indicate that new states are indeed formed upon adsorption. A further sign of the modification of the molecular electronic structure of the TPA/Au(111) is the disappearance of the shake-up feature in the C 1s photoemission spectrum (Figure S2). This indicates that adsorption induces a modification of the empty DOSs involved in the shake-up process. This will be further discussed together with the calculated DOSs of TPA/Au(111). A significant electronic coupling between TPA and Au(111) is also supported by the energy positions of the N 1s and C 1s PES lines (marked by black bars in the NEXAFS spectra in Figure 2a,b), which are found at the edge of the absorption spectrum (see also Figure S3). This can be understood considering that upon chemisorption the surface provides an efficient screening of the core–hole created by photoemission, so that the PES BE can be regarded as the Fermi level of the empty valence states.^19^ This is useful for allowing an alignment of the filled and empty valence states to look closer at what happens to the DOS in the original molecular gap.

### Valence Band Photoemission Results

**Figure 3:**
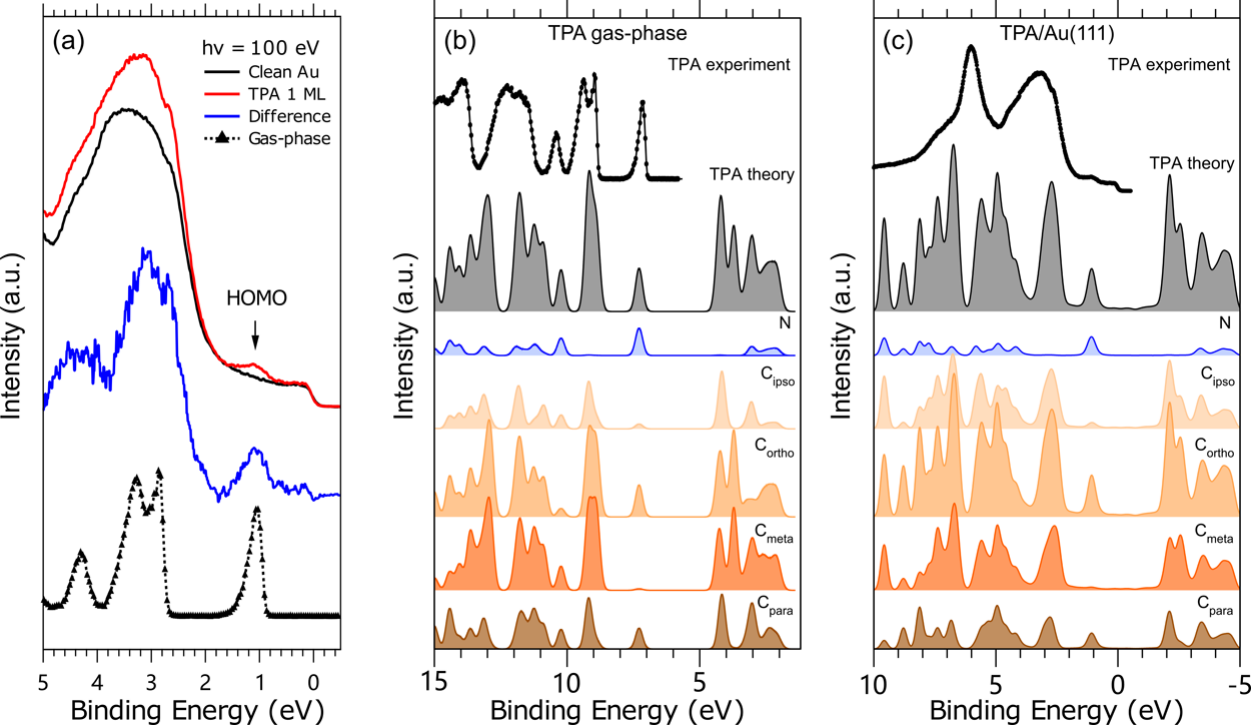
(a) Comparison between the valence PE spectra measured at 100 eV of TPA adsorbed on Au(111) (red line) and of TPA in the gas phase (black line with markers). The gas-phase spectrum was shifted by −6.1 eV to align with the solid-state spectrum. The contribution of TPA to the spectrum of TPA/Au(111), blue curve, that was obtained by subtracting the clean Au(111) spectrum from that of TPA/Au(111) is also shown. (b) Theoretical total DOS of TPA in the gas phase (gray curve) and partial DOS (pDOS) of each type of C and N atoms. The DOS and pDOS are aligned with the HOMO of the experimental valence PES (black curve with markers) by a shift of 6.39 eV. (c) Theoretical partial DOS of TPA for TPA/Au(111) (gray curve, after subtracting the contribution from the Au atoms) and pDOS of each type of C and N atoms. The pDOS are aligned with the HOMO of the experimental valence PES (black curve) by a shift of 0.85 eV.

As shown in Figure 3a, due to the low coverage of TPA, the valence band photoemission results for clean Au and for TPA/Au(111) are very similar and dominated by the gold valence structure. After performing a subtraction of the clean Au(111) (black curve) from the adsorbate valence band spectrum (red curve), we got a difference curve (blue curve), which we further compared to the valence PE spectrum of gas-phase TPA (black curve with markers). All spectra were measured at 100 eV photon energy. The obtained difference spectrum compares well to the gas-phase TPA molecular valence results. The subtraction allows us to find the HOMO of the adsorbed TPA at a BE of 1.05 eV to which we align the gas-phase spectrum (Figure 3a).

To better understand the origin of the NEXAFS pre-edge peak and to analyze the whole adsorption interaction of TPA on Au(111), we have computed the DOS of the free molecule and of the adsorbed system in the ground state (i.e., without core–hole), as shown in Figure 3b,c, respectively. The theoretical spectra are shifted by 6.39 and 0.85 eV, respectively, to match the experimental energy scales. The computed DOS includes a portion of the unoccupied region from about 5 eV for the gas phase and from about 1.5 eV for the adsorbed TPA. We also report the total DOS of the molecule and the partial DOSs related to the contributions of the N and C atoms with respect to the experimental valence spectra for the gas-phase and adsorbed TPA. The overall profiles of the DOS of the TPA in the gas phase and adsorbed on Au(111) are very similar, and the relative intensities and distances between the main peaks are comparable, as can be seen from the partial DOS of the N and C atoms. A more detailed inspection of the computed DOS of TPA/Au(111) (Figure 3c) shows that the main occupied N peak (TPA HOMO) is located at 1.05 eV, while the main unoccupied N peak lies at about −3.4 eV. At about −2 and at −3 eV, we find the main unoccupied C peaks having contributions from C-*meta*, C-*para*, and C-*ortho*.

Evidently, a striking difference between the calculated DOS of free and adsorbed TPA is found in the HOMO–LUMO gap region. In the theoretical DOS of the adsorbed TPA, new electronic states of the low intensity populate the region extending from the HOMO to −2 eV, that is, the energy region corresponding to the HOMO–LUMO gap of the gas phase. This can be clearly seen in the DOS of the TPA represented as a gray filled curve in Figure 3c. These results give a strong indication that these empty electronic states of N and C characters are formed because of the adsorption of TPA on the gold surface. Consequently, the hybridization between the gold and the molecular states can give rise to the pre-edge features observed in the C and, more clearly, in the N K-edge NEXAFS spectra shown in Figure 2b. These theoretical and experimental results indicating the creation of new states due to the interaction of the TPA molecules with the Au surface seem to be in contradiction with the belief that gold is an inert metal. The surface shows to maintain the herringbone reconstruction upon adsorption (see also STM results in Figure 1) usually considered a sign of a weak molecular substrate interaction. However, a recent DFT work^20^ on the Au(111) surface reconstruction enlightened that the herringbone structure can promote more reactive surface sites for molecular adsorption.

### Energy Level Alignment

To further understand experimentally the significance of the pre-edge feature, we performed an energy level alignment analysis of the core-hole final state by applying a method introduced by Schnadt et al.^21^ and discussed in detail by Brühwiler et al.,^22^ using the photoemission valence and NEXAFS results. For the adsorbate system, we have considered the core levels PES BE (Figures S2 and S3) as the Fermi edge of the valence empty states,^19^ as previously discussed. According to this method, the energy level alignment provides an overview of the occupied (valence and PES) and unoccupied DOSs in the presence of the core hole (NEXAFS).

**Figure 4:**
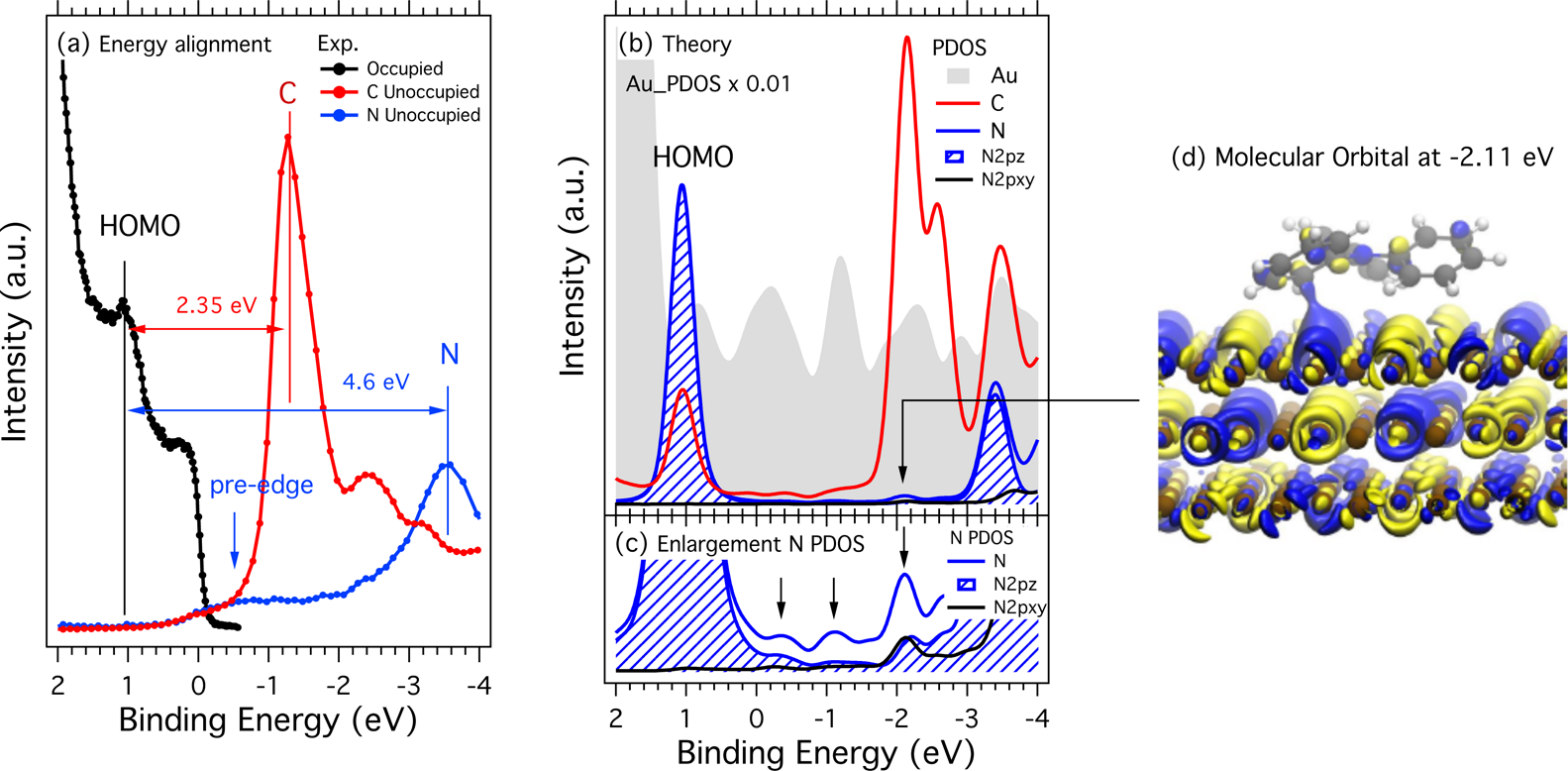
(a) Energy alignment of experimental valence photoemission and NEXAFS data (core–hole final state). (b) Computed ground-state partial DOS of N, N p_*xy*_, and N p_*z*_, with C and Au aligned with the experimental HOMO; (c) same as (b) but enlarged in the vertical axis; and (d) iso-surface of the electronic state that lies in the N pre-edge region at −2.11 eV.

The energy level alignment for TPA/Au(111) is shown in Figure 4a. The energy difference between the HOMO and the first unoccupied state of carbon of significant intensity, at 2.35 eV, is very close to a HOMO–LUMO gap of 2.36 eV measured for gas-phase TPA.^6^ However, the energy separation between the HOMO and the main N unoccupied state is reduced to 4.6 eV, as compared to a gas-phase value of 5.6 eV.

Moreover, the pre-edge feature of the N and C unoccupied states appears to extend over the previous HOMO–LUMO gap of the free TPA and overlap with the Au Fermi level. This is the experimental evidence that the HOMO–LUMO gap gets filled upon adsorption in the core-hole final state. To understand if these new states are the results of the core excitation (final state effect) or if they are instead the result of the adsorption process (initial state effect), we show in Figure 4 the energy level alignment and the calculated ground-state DOS of TPA/Au(111).

Clearly, one cannot expect equivalence in the energy position of the peaks in Figure 4a,b because they depict the experimental PES and NEXAFS spectra with core-hole and the calculated DOS in the ground state, respectively. Nevertheless, very interestingly, qualitative analogies in the structure of the experimental and theoretical curves are observed. In all the pDOS of Figure 4b, we can clearly discern the new electronic states that populate the BE window between 1.05 eV (HOMO of TPA, mainly of N character) and −1.3 eV (LUMO of TPA, mainly of the C character). The resulting picture is that the new unoccupied molecular states observed in the DOS upon adsorption are the targets of the electron transitions in the photon absorption (NEXAFS) process and are detected as the pre-edge feature.

In the enlargement in Figure 4c, it is clearly seen that the three main new C and N peaks at −0.35, −1.10, and −2.11 eV are aligned with the Au peaks in the same region, and therefore we can conclude that the pre-edge feature seen in the NEXAFS spectra of TPA/Au(111) occurs by the hybridization between the molecular orbitals and the metal electronic states. Due to the adsorption orientation of the molecules, these orbitals also have some in-plane components.

Finally, to look closer at the new states, we show in Figure 4d a picture of an iso-surface corresponding to the electronic state that lies in the N pre-edge region at −2.11 eV. The state is a mix of TPA and (mainly) Au states, and it extends in the space between the surface and the molecules, mostly localized on the Au surface. It has a strong out-of-plane component with respect to the Au surface. However, because it also involves a C-*meta* atom in the phenyl ring, which is slightly inclined with respect to the Au surface that defines our *xy* plane, it also has in-plane components.

The present study contributes to the understanding of the molecular–surface interaction for weakly adsorbed systems. In a previous study of the adsorption of another organic molecule, namely, the metal free phthalocyanine, on Au(111) with the very same combination of experimental and computational techniques, we reported analogous results, indicating the occurrence of a similar kind of adsorption mechanism for metal-free phthalocyanine on Au(111).^23^ Moreover, hybrid substrate-molecular states were also both observed and calculated at the interface between a boroxine molecule and the Au(111) surface.^24^ The main implication of our results is that the adsorption of TPA on gold induces strong modifications in the electronic structure of the molecule, specifically its HOMO–LUMO gap. This means that the molecules adsorbed on the Au(111) surface lose the properties they have in the gas phase or in thick films with the appearance of new electronic states filling the HOMO–LUMO gap. Strong evidence of a significant interaction between the molecules and the surface is also confirmed by the disappearance of the shake-up feature in the C 1s photoemission spectrum (Figure S2) upon adsorption. This result indicates a modification of the valence electronic structure of TPA when it is adsorbed on the surface with a quenching/modification of the possible shake-up transitions. As observed in the figure, the C 1s shake-up of the gas-phase TPA at about 7 eV seems smeared out and the shake-up closest to the C 1s line at 2.45 eV from the main line, assigned to HOMO–LUMO transitions in our previous study,^6^ is now hidden within the background tail of the weaker peak (marked with a red bar). Such a background can be related to shake-up transitions from states just below the Fermi level to empty states just above Fermi, as observed for C 1s PE spectra of organic monolayer films on other metal surfaces.^25−29^ In our case, our theoretical calculations support the latter scenario, indicating the existence of new states in the original HOMO–LUMO gap that are now available for the shake-up transitions. These kinds of results are often related to quite strong interactions, like those occurring in chemisorbed systems,^17,18^ as confirmed by our experimental NEXAFS measurements where the energy position of the N 1s and C 1s PES lines are found at the edge of the absorption spectrum.

## Conclusions

A monolayer of TPA/Au(111) was characterized by core and valence PES and NEXAFS spectroscopy and STM. Through the comparison with our previous investigations of TPA in the gas phase^5^ and ab initio calculations, we could shed light on the considerable electronic structure modifications connected to the molecule–surface interactions. We show that upon adsorption on Au(111), new molecular states appear in the energy region that was the HOMO–LUMO gap of TPA in the gas phase. These states can be directly detected by means of NEXAFS spectroscopy, where they appear as a broad pre-edge peak in the N K-edge also reproduced by our ab initio simulations of the NEXAFS spectra. The feature is also visible in the C K-edge NEXAFS, and the presence of states between the molecule and the substrate is further confirmed by the ground-state DOS calculations. From an experimental point of view, we can clearly see the new states at the N K-edge NEXAFS because of the large energy gap between the HOMO and the first N intense absorption transition, whereas this feature is hidden by the high-intensity π* resonance in the C K-edge NEXAFS spectrum.

The partial DOS calculations performed for the adsorption system in the ground state indicate that when TPA is adsorbed on Au(111) new low-intensity empty states become available in the HOMO–LUMO gap of the molecule. Hence, the pre-edge feature that appears in the N K-edge NEXAFS spectrum has an initial state character connected to the bonding between the TPA molecule and the Au(111).

However, the distances obtained by the calculations reveal a system where the interaction between the molecules and the Au(111) surface is rather weak. Hence, final state effects could still play a role even in the TPA case, although, according to our results, they do not provide the full explanation to the observed N K-edge NEXAFS spectra. These new empty states are, moreover, aligned with the peaks of the gold partial DOS in the same region, proving that the pre-edge peak measured in the NEXAFS is due to transitions into new hybrid states created upon the adsorption process, that is, an initial state effect, and coupled to the electronic structure of the adsorption system without, however, excluding possible final state effects. This also implies that the adsorption of TPA on Au(111) does not have the character of a physisorption because here the perturbation of the electronic states is considerable.

To conclude, the most dramatic effect in the adsorption of TPA on Au(111) is the huge reduction or even disappearance of the HOMO–LUMO gap of the molecule, a fact that is crucial for a wide range of possible technological applications based on TPA and gold. Because Au(111) is generally considered as a low-reactive surface and the computed adsorption distances for TPA would suggest a rather low interaction between the molecule and the substrate, this fact may be unexpected. Our study emphasizes that the precise electronic modifications exerted on organic molecules by the adsorption on metallic surfaces are not yet understood. Our results demonstrate the occurrence of a molecule-metal contact built through TPA–Au hybrid states, which might have an impact on the charge mobility through the substrate. On the other hand, the filling of the molecular energy gap would compromise the optical absorption and the exciton creation in solar cell applications. However, our results also suggest that only the TPA–Au interface is affected by the molecule–metal hybrid states, whereas thicker films of TPA keep a molecular character, which could still imply the possibility to use TPA in 2D optoelectronic applications. It is also worth noting that although the TPA molecule could function as the donor component in many optoelectronic components, it is not often implemented in such devices due to its low desorption temperature. Instead, larger molecules with TPA as a building block (e.g., *m*-MTDATA and DTDCTB molecules) are usually preferred.

## Supporting Information Available

N K-edge NEXAFS for different coverages of TPA/Au(111); C 1s and N 1s PES results; adsorption energy of TPA/Au(111); and individual C contributions to C 1s NEXAFS (PDF)
