# Supplementary material for: Clarifying the Adsorption of Triphenylamine on Au(111): Filling the HOMO–LUMO Gap
Source: J Phys Chem C Nanomater Interfaces. 2022 Jan 17;126(3):1635–43. doi: 10.1021/acs.jpcc.1c08877 (PMC8802676; doi:10.1021/acs.jpcc.1c08877)
Supplement: Supplementary file 1 — jp1c08877_si_001.pdf [file jp1c08877_si_001.pdf]

Supporting Information

Clarifying the Adsorption of Triphenylamine on Au(111): Filling the HOMO-LUMO Gap

Teng Zhang,[1,] Pamela H.W. Svensson,[2] Iulia Emilia Brumboiu,[3] Valeria Lanzilotto,[4] Cesare Grazioli,[5] Ambra Guarnaccio,[6] Fredrik O.L. Johansson,[2,7,8] Klára Beranová,[9,10] Marcello Coreno,[6] Monica de Simone,[5]Luca Floreano,[5] Albano Cossaro,[5,11] Barbara Brena,[2,*] and Carla. Puglia[2,*]*

[1] School of Integrated Circuits and Electronics, MIIT Key Laboratory for Low-Dimensional Quantum Structure and Devices, Beijing Institute of Technology, 100081 Beijing, China;

[2] Department of Physics and Astronomy, Uppsala University, Box 516, SE-751 20 Uppsala, Sweden;

[3] Department of Chemistry, Pohang University of Science and Technology (POSTECH), 37673 Pohang, Republic of Korea;

[4] Sapienza Università di Roma, Department of Chemistry, P.le A. Moro 5, 00185 Roma, Italy;

[5] IOM-CNR, Laboratorio TASC, Sincrotrone Trieste, Basovizza, Trieste, Italy;

[6] ISM-CNR, Istituto di Struttura della Materia, 85050 Tito Scalo (Pz), Italy;

[7] KTH Royal Institute of Technology, Division of Applied Physical Chemistry, Department of Chemistry, 10044 Stockholm, Sweden;

[8] Sorbonne Université, UMR CNRS 7588, Institut des Nanosciences de Paris, F-75005 Paris, France.

[9] Elettra-Sincrotrone Trieste S. C. p. A., Strada Statale 14, km 163.5, Basovizza, 34149 Trieste, Italy;

[10] FZU – Institute of Physics of the Czech Academy of Sciences, 18221 Prague, Czech Republic;

[11] Department of Chemical and Pharmaceutical Sciences, University of Trieste, 34127 Trieste, Italy;

*Corresponding authors: teng.zhang@bit.edu.cn; barbara.brena@physics.uu.se;carla.puglia@physics.uu.se

N Kedge NEXAFS for different coverages of TPA/Au(111)

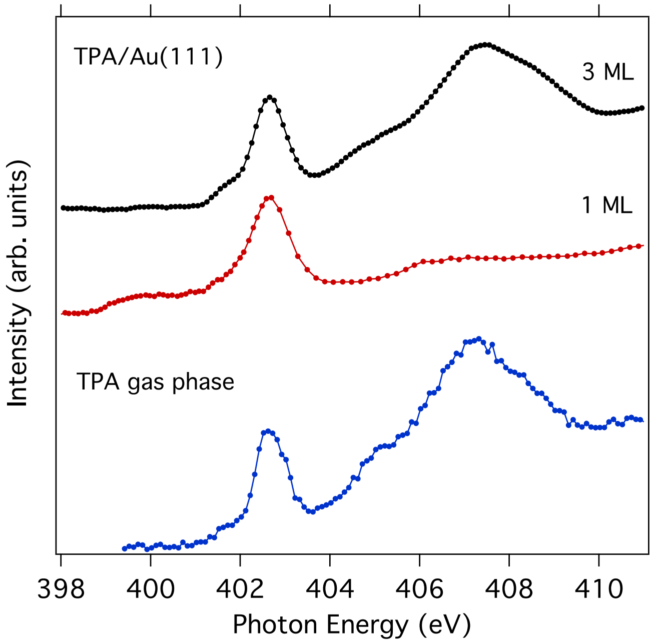

Figure S1 N K-edge NEXAFS spectra of TPA in gas-phase, of one monolayer (1 ML) and three layers (3 ML) of TPA adsorbed on Au(111). The pre-edge feature is clearly visible in the 1 ML spectrum but disappears in the 3 ML spectrum, indicating that it is the result of the interaction between the molecules and the surface.

C 1s and N 1s Photoelectron Spectroscopy

In Figure S2(a), we can observe a main peak at a BE of 283.82 eV and a weaker peak at the higher BE of 285.02 eV. In our previous study of gas-phase TPA,[1] we could attribute the lower intensity peak, that in gas-phase was located at 290.94 eV BE, to the C-*ipso,* while the main peak at the lower BE of 289.85 eV, to all the other C atoms (C-*ortho*, C-*meta* and C-*para* atoms). The adsorbed TPA spectrum profile is very similar to the gas-phase one. The general broadening of the adsorbate spectrum represents the main difference due to solid state effects,[2-3] therefore we maintain here the same assignment. The red and black arrows in the Figure S2(a) point to the shake-up features of TPA in gas-phase and /Au(111), respectively.

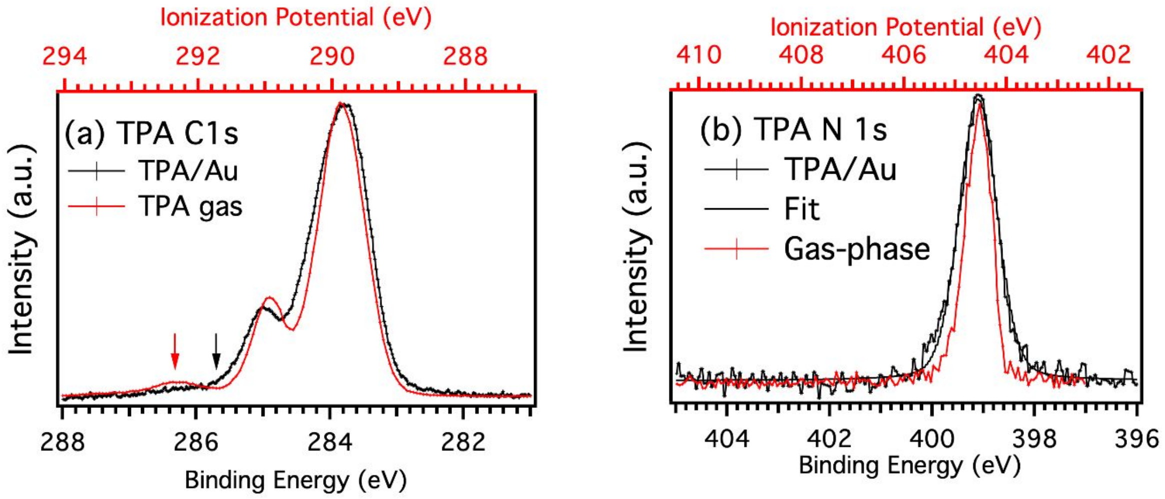

Figure S2: The (a) C 1s and (b) N 1s photoemission spectra showing comparison between gas-phase and 1 ML TPA/Au(111). Red and black arrows in (a) point to the shake-up in gas-phase and the C 1s tail in TPA/Au(111).

In Figure S2(b) we present the N 1s PE spectrum of 1 ML TPA/Au(111) in comparison to our previous gas-phase result.[1] The spectrum can be fitted by only one spectral component found at 399.11 eV. As for the C 1s PE spectrum and for similar reasons, the N 1s shows a broadening compared to the gas-phase results (FWHM 0.88 *vs* 0.56 eV) and a small asymmetry on the higher binding energy side of the peak.

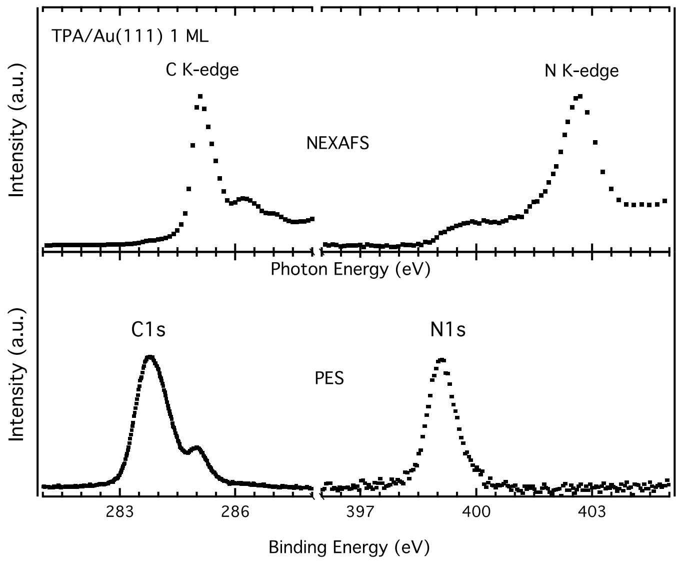

Figure S3: C1s and N1s core lines in (top) photoabsorption and (bottom) photoemission for 1 ML TPA/Au(111).

Adsorption Energy of TPA/Au(111)

The adsorption energy was computed as the difference between the total energy of the adsorbed system (TPA+surface) and the sum of the total energies of the molecule and of the surface and results in -2.00 eV. Since we do not have an experimental reference and, moreover, the calculated adsorption energy strongly depends on the choice of the DFT functionals and of the van der Waals descriptions,[4] this result is not a reliable parameter to evaluate the strength of the bonding of the molecule on the surface.

The propeller-like conformation of the TPA does not allow to simply evaluate the binding energy per each C atom. As the molecule has a non-flat geometry, there are six C atoms which are near the Au surface, with distances around 3.1 Å from their closest Au atoms. This would give a maximum value of 330 meV per each of these atoms, which would be rather high. However, this excludes all the other C atoms that are in the molecule, so in principle the binding energy for the C atoms closer to the surface could be lower.

Individual C contributions to C1s NEXAFS

Figure S4 shows the individual contributions in-plane and out-of-plane to the C K-edge NEXAFS in the HOL2 configuration of each C atom separately: C_{ipso}, C_{ortho} , C_{meta} (where *far* and *close* indicate the C_{ortho} or C_{meta} atoms closer or farther from the Au surface) and C_{para}.

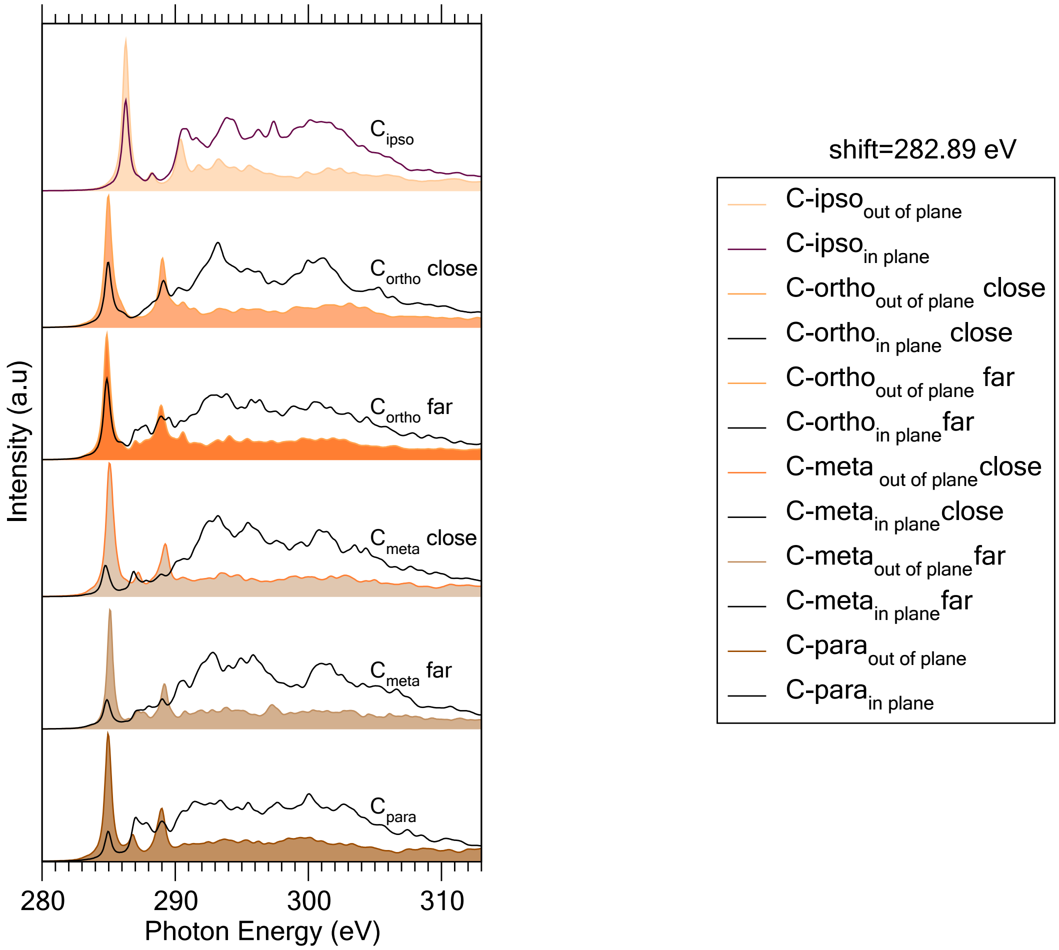

Figure S4: Individual contributions to the C K edge NEXAFS in the HOL2 configuration of each C atom separately.

References

1. Zhang, T., et al., Lone-Pair Delocalization Effects within Electron Donor Molecules: The Case of Triphenylamine and Its Thiophene-Analog. *J Phys Chem C* 2018, *122*, 17706-17717.

2. Bidermane, I., et al., Characterization of Gas Phase Iron Phthalocyanine with X-Ray Photoelectron and Absorption Spectroscopies. *physica status solidi (b)* 2015, *252*, 1259-1265.

3. Tillborg, H.; Nilsson, A.; Mrtensson, N., Shake-up and Shake-Off Structures in Core Level Photoemission Spectra from Adsorbates. *Journal of Electron Spectroscopy and Related Phenomena* 1993, *62*, 73-93.

4. Lüder, J.; Sanyal, B.; Eriksson, O.; Puglia, C.; Brena, B., Comparison of Van Der Waals Corrected and Sparse-Matter Density Functionals for the Metal-Free Phthalocyanine/Gold Interface. *Physical Review B* 2014, *89*, 045416-045416.
